# Supplementary material for: Chlorella pyrenoidosa Polysaccharide CPP-3a Promotes M1 Polarization of Macrophages via TLR4/2-MyD88-NF-κB/p38 MAPK Signaling Pathways
Source: Mar Drugs. 2025 Jul 16;23(7):290. doi: 10.3390/md23070290 (PMC12299851; doi:10.3390/md23070290)
Supplement: Supplementary file 1 [file marinedrugs-23-00290-s001.zip › marinedrugs-3751825-supplementary.pdf]

Supplementary data

1. Cell viability assay

RAW264.7 cells were seeded in 96-well plates at a density of 1×10^4 cells/well and cultured overnight. Cells were then treated with CPP-3a (100, 10, or 1 μg/mL) or LPS (1 μg/mL) for 72 hours. Cell proliferation was assessed using a Cell Counting Kit-8 according to the manufacturer's instructions.

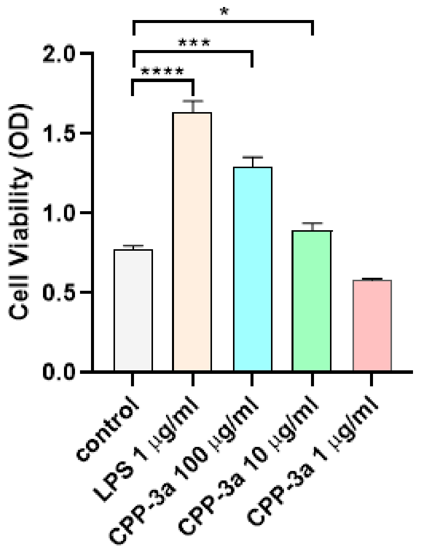

Supplementary Figure S1: Cell viability of RAW264.7 cells treated with LPS or CPP-3a for 72 hours.
